# Smartphone addiction, physical activity engagement, and sleep quality in Chinese adolescents: a parallel and serial mediation model

**DOI:** 10.3389/fpsyt.2026.1966617

**Published:** 2026-09-29

**Authors:** Weidong Zuo, Bing Zhang

**Affiliations:** School of Physical Education, Huanggang Normal University, Huanggang, China

**Keywords:** adolescents, depressive symptoms, physical activity, serial mediation, sleep quality, smartphone addiction

## Abstract

**Introduction:**

Excessive smartphone use has become a major public health concern among adolescents, particularly in China where smartphone use is widespread. Although smartphone addiction is consistently associated with poor sleep quality, the mechanisms underlying this association remain insufficiently understood. This study examined whether physical activity engagement and depressive symptoms operate as parallel and serial mediators linking smartphone addiction to sleep quality in Chinese adolescents.

**Methods:**

A cross-sectional survey was conducted among 612 adolescents (Mage = 14.2 years, SD = 1.6; 51.3% female) from four secondary schools in Hubei Province, China. Participants completed the Smartphone Addiction Scale–Short Version, the Physical Activity Questionnaire for Adolescents, the Pittsburgh Sleep Quality Index, and the Depression subscale of the Depression Anxiety Stress Scales-21. Path analysis of observed composite scores with robust maximum likelihood estimation and bias-corrected bootstrapping (5,000 resamples) was used to test the proposed parallel and serial mediation model.

**Results:**

Smartphone addiction was positively associated with poor sleep quality (β = .38, p <.001). Both physical activity (indirect effect = .04, 95% CI [.01,.07]) and depressive symptoms (indirect effect = .14, 95% CI [.09,.19]) significantly mediated this association in parallel. In addition, the serial pathway from smartphone addiction to low physical activity, elevated depressive symptoms, and poor sleep quality was significant (indirect effect = .02, 95% CI [.01,.04]). The mediators collectively accounted for 52.6% of the total effect.

**Discussion:**

These findings suggest that problematic smartphone use is associated with poorer adolescent sleep through behavioral and psychological pathways involving reduced physical activity and elevated depressive symptoms. School-based interventions that promote physical activity while reducing problematic smartphone use may help protect adolescent sleep and mental health. Longitudinal and experimental designs are needed to establish causal direction and inform intervention development.

## 1 Introduction

The rapid proliferation of smartphones has transformed the daily lives of adolescents worldwide, with concerns growing about excessive screen time and its potential health consequences (1, 2). While smartphones offer educational and social benefits, excessive use has been linked to a range of negative outcomes, including sleep disturbances, depressive symptoms, anxiety, and reduced physical activity (3, 4). Understanding the mechanisms through which problematic smartphone use is associated with adolescent well-being is therefore of considerable public health importance.

A robust body of research has established a link between smartphone addiction and poor sleep quality. Systematic reviews have shown that screen time, particularly before bedtime, is associated with shortened sleep duration, prolonged sleep latency, and reduced sleep efficiency in school-aged children and adolescents (1). The mechanisms proposed include physiological (blue light exposure suppressing melatonin), psychological (cognitive arousal from content), and behavioral (displacement of sleep time) pathways (2). However, most studies have examined the direct smartphone–sleep relationship, leaving the intermediate pathways insufficiently understood.

Physical activity represents one potential mediator in this relationship. The displacement hypothesis suggests that time spent on smartphones may be associated with reduced time available for physically active pursuits (5). Indeed, prior studies have examined physical activity and smartphone use jointly in relation to sleep quality among Chinese college students (6), and one study tested a chain mediation from physical activity to sleep quality via stress and smartphone addiction during the COVID-19 pandemic (7). A longitudinal study in Chinese adolescents further demonstrated that mobile phone addiction longitudinally predicted reduced physical activity, which in turn predicted sleep procrastination (8). Physical activity, in turn, is well established as a protective factor for sleep quality. Regular exercise is associated with improved sleep duration, efficiency, and quality across the lifespan (9, 10). Furthermore, physical activity has been shown to buffer against stress and reduce depressive symptoms (11, 12), providing a plausible link between smartphone use, mental health, and sleep.

Depressive symptoms represent a second potential mediator. Excessive smartphone use has been associated with increased depressive symptoms in multiple longitudinal studies (4, 13). Depression, in turn, is one of the most robust predictors of poor sleep quality, characterized by sleep onset insomnia, fragmented sleep, and daytime dysfunction (14). Prior research has demonstrated that depression mediates the relationship between problematic mobile phone use and sleep quality among Chinese adolescents (15). However, prior studies have examined these constructs primarily in college student populations (6, 7) or have focused on sleep procrastination rather than sleep quality (8), and no study to our knowledge has simultaneously examined physical activity and depressive symptoms as parallel and serial mediators of the smartphone addiction–sleep quality link in adolescents. Testing both pathways in a single model would provide a more comprehensive understanding of the mechanisms involved.

The stress buffering hypothesis (16) provides a theoretical framework for the current study. According to this model, physical activity may serve as a coping resource that buffers the impact of stressors (including digital stress from smartphone addiction) on mental health and sleep. Additionally, self-determination theory (17, 18) posits that fulfilling basic psychological needs through intrinsically motivated behaviors such as physical activity promotes well-being and protects against psychopathology. When smartphone addiction is accompanied by lower physical activity, these psychological needs may remain unfulfilled, which is in turn associated with depressive symptoms and poorer sleep.

Building on these theoretical and empirical foundations, the present study aimed to test a parallel and serial mediation model (**Figure 1**) in which: (a) smartphone addiction is associated with reduced physical activity and elevated depressive symptoms; (b) reduced physical activity and elevated depressive symptoms are independently associated with poor sleep quality (parallel mediation); and (c) the effect of smartphone addiction on sleep quality is serially mediated through physical activity and then depressive symptoms (smartphone addiction → low physical activity → depressive symptoms → poor sleep quality). We hypothesized that all three pathways would be significant, providing a more nuanced understanding of how smartphone addiction is associated with adolescent sleep.

**Figure 1:**
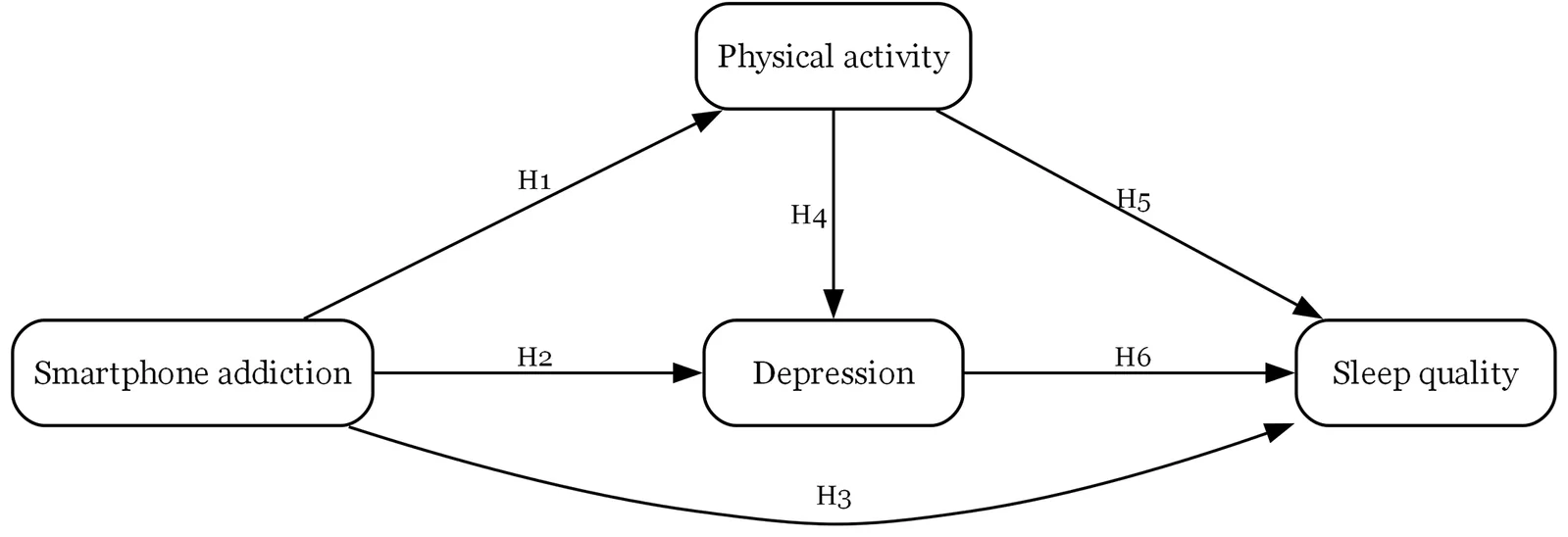
The hypothesized parallel and serial mediation model. Solid arrows represent hypothesized direct effects; the dashed arrow represents the hypothesized serial mediation pathway via reduced physical activity and elevated depressive symptoms.

The Chinese context provides a particularly relevant setting for this investigation. Smartphone use is widespread among Chinese secondary school students, and the academic pressure faced by these adolescents, combined with limited leisure time, makes the potential displacement of physical activity by smartphone use especially concerning. Understanding how smartphone addiction, physical activity, and mental health intersect to influence sleep in this population is critical for developing culturally appropriate interventions.

Despite the growing literature on smartphone use and adolescent health, several gaps remain. First, most prior studies have examined bivariate relationships (e.g., smartphone use and sleep, or smartphone use and depression) without considering multiple mediating pathways simultaneously. Second, physical activity is rarely included as a mediator in models linking digital behaviors to sleep outcomes, despite its well-documented benefits for both mental health and sleep. Third, the serial pathway from smartphone addiction through physical activity to depressive symptoms and then to sleep quality has not been tested, limiting our understanding of the interrelated pathways linking digital behaviors to health. The present study addresses these gaps by testing a comprehensive parallel and serial mediation model in a Chinese adolescent sample.

## 2 Materials and methods

### 2.1 Participants and procedure

A cross-sectional survey design was employed. Participants were recruited from four secondary schools in Huanggang City, Hubei Province, China, using convenience sampling (School A, n = 168; School B, n = 156; School C, n = 149; School D, n = 139). Data collection took place between September and November 2024. A total of 680 questionnaires were distributed, and 612 valid responses were obtained, yielding a response rate of 90.0%. Questionnaires were excluded for incomplete responses (n = 45) or patterned/straight-line responding (n = 23); all 612 valid questionnaires had complete item-level data. Inclusion criteria were: (a) enrolled in grades 7–12, (b) owned a smartphone, and (c) provided informed assent. Exclusion criteria were: (a) presence of diagnosed sleep disorders or (b) current use of psychotropic medications. The final sample consisted of 612 adolescents (314 females, 51.3%; 298 males, 48.7%) with a mean age of 14.2 years (SD = 1.6, range = 12–18). The majority (78.4%) lived with both parents, and 43.2% resided in rural areas. The study was approved by the Ethics Committee of Huanggang Normal University. Written informed consent was obtained from parents/guardians and assent from adolescents. All procedures were in accordance with the Declaration of Helsinki.

### 2.2 Measures

Smartphone Addiction Scale–Short Version (SAS-SV). The SAS-SV (19) is a 10-item self-report measure of smartphone addiction severity. Items are rated on a 6-point Likert scale (1 = strongly disagree to 6 = strongly agree), with total scores ranging from 10 to 60. Higher scores indicate greater smartphone addiction severity. The SAS-SV is a self-report screening instrument for problematic smartphone use severity rather than a clinical diagnostic tool (20), and it assesses general severity without a specific recall timeframe. The Chinese version of the SAS-SV has demonstrated acceptable psychometric properties in adolescent populations. In the present study, Cronbach’s alpha was.88.

Physical Activity Questionnaire for Adolescents (PAQ-A). The PAQ-A (21) is an 8-item self-administered measure of physical activity engagement over the past seven days. Items assess participation in various physical activities, including sports, games, and active transportation. The PAQ-A yields a summary score ranging from 1 to 5, with higher scores indicating greater physical activity. The Chinese version has shown acceptable psychometric properties in adolescents. In the present study, Cronbach’s alpha was.82.

Pittsburgh Sleep Quality Index (PSQI). The PSQI (22) is a 19-item self-report measure assessing sleep quality over the past month. The measure yields seven component scores: subjective sleep quality, sleep latency, sleep duration, habitual sleep efficiency, sleep disturbances, use of sleeping medication, and daytime dysfunction. The components are summed to produce a global score ranging from 0 to 21, with higher scores indicating poorer sleep quality. A global score >5 indicates poor sleep quality. The Chinese version of the PSQI has demonstrated acceptable psychometric properties. In the present study, Cronbach’s alpha was.84.

Depression Anxiety Stress Scales-21 (DASS-21). The DASS-21 (23) is a 21-item self-report measure of depressive, anxiety, and stress symptoms. Items are rated on a 4-point Likert scale (0 = did not apply to me to 3 = applied to me very much or most of the time), referring to the previous week. The depression subscale (7 items) was used in the present study to assess depressive symptom severity; the anxiety and stress subscales were not administered. The Chinese version has demonstrated acceptable psychometric properties in adolescent populations. In the present study, Cronbach’s alpha for the depression subscale was.85.

### 2.3 Data analysis

Descriptive statistics, bivariate correlations, and internal consistency coefficients were computed using SPSS 26.0 (24). Path analysis was conducted using Mplus 8.3 (25) with robust maximum likelihood estimation (MLR) to account for non-normality. The analysis used observed composite scores (SAS-SV total, PAQ-A summary score, DASS-21 depression subscale sum, PSQI global score) rather than latent variables with multiple indicators; because each construct is represented by a single observed indicator, a separate confirmatory factor analysis step is not applicable. Because all directional paths among the four focal variables were estimated, the focal path model is just-identified (zero degrees of freedom); global fit indices are therefore not informative and are not reported. Model evaluation focuses on the magnitude and significance of individual path estimates and on bootstrap tests of the indirect effects, consistent with regression-based mediation practice (26). Multicollinearity was assessed via variance inflation factor (VIF) and tolerance statistics; VIF values ranged from 1.04 (residential area) to 1.28 (depressive symptoms) and tolerance values from.78 to.96, all within acceptable limits.

The hypothesized model included smartphone addiction as the predictor, physical activity and depressive symptoms as parallel mediators, and sleep quality as the outcome. The model was specified *a priori* based on theory; no *post hoc* modifications were made and no residual correlations were estimated. Age, gender, and residential area were included as covariates: age because smartphone use, physical activity, and sleep change considerably across adolescence, gender because of established differences in smartphone use and depressive symptoms, and residential area because of urban–rural differences in activity opportunities and academic context (1, 4); each covariate was also correlated with at least one study variable in the present sample (**Table 1**). Indirect effects were tested using bias-corrected bootstrapping with 5,000 resamples. Indirect effects were deemed significant when the 95% confidence interval (CI) did not include zero (26). Common method bias was examined using Harman’s single-factor test as a limited diagnostic only; no single factor accounted for more than 35.6% of the variance, below the 50% threshold, although this test is known to be insensitive and cannot rule out common method bias.

**Table 1:** Descriptive statistics and bivariate correlations of study variables.

Variable	M	SD	1	2	3	4
1. Smartphone addiction	31.5	9.8	—			
2. Physical activity	2.7	0.7	-.25***	—		
3. Depressive symptoms	8.9	5.4	.45***	-.34***	—	
4. Sleep quality (PSQI)	6.8	3.2	.42***	-.28***	.51***	—
5. Age	14.2	1.6	.12*	-.08	.15**	.10*
6. Gender (1=F, 0=M)	—	—	.09	-.11*	.08	.07
7. Rural (1=rural)	—	—	.06	-.14**	.10*	.08

N = 612. PSQI, Pittsburgh Sleep Quality Index (higher scores indicate poorer sleep). *p <.05, **p <.01, ***p <.001 (two-tailed).

### 2.4 Power analysis

*A priori* power analysis using G*Power 3.1 was conducted as a regression-based approximation. Because exact power analysis for complex mediation models requires Monte Carlo simulation, we approximated power using the largest single regression equation in the model (sleep quality regressed on smartphone addiction, physical activity, depressive symptoms, and the three covariates). The analysis indicated that a sample of approximately 150 would achieve statistical power of.95 to detect a medium effect size (f² = .15) in multiple regression with six predictors at α = .05. The obtained sample of 612 exceeded this minimum requirement. We acknowledge that this approximation does not guarantee adequate power for detecting small indirect effects, particularly the serial pathway, and this point is revisited in the limitations.

## 3 Results

### 3.1 Descriptive statistics and correlations

Descriptive statistics and bivariate correlations are presented in **Table 1**. The mean SAS-SV score was 31.5 (SD = 9.8), with 22.1% of participants exceeding the cutoff for smartphone addiction (boys: >31; girls: >33). The mean PAQ-A score was 2.7 (SD = 0.7), indicating moderate physical activity levels. The mean PSQI score was 6.8 (SD = 3.2), with 46.2% of participants classified as poor sleepers (PSQI > 5). The mean DASS-21 depression subscale score was 8.9 (SD = 5.4).

Smartphone addiction was positively correlated with poor sleep quality (r = .42, p <.001) and depressive symptoms (r = .45, p <.001), and negatively correlated with physical activity (r = -.25, p <.001). Physical activity was negatively correlated with poor sleep quality (r = -.28, p <.001) and depressive symptoms (r = -.34, p <.001). Depressive symptoms were positively correlated with poor sleep quality (r = .51, p <.001).

### 3.2 Path analysis

The hypothesized parallel and serial mediation model was estimated as specified. Because all directional paths among the four focal variables were estimated, the model is just-identified (df = 0) and global fit indices are not informative; the results therefore focus on the path estimates and bootstrap tests. As shown in **Figure 2**, all hypothesized paths were significant. Smartphone addiction was positively associated with depressive symptoms (β = .41, p <.001), negatively associated with physical activity (β = -.22, p <.001), and positively associated with poor sleep quality (β = .19, p = .003). Physical activity was negatively associated with depressive symptoms (β = -.26, p <.001) and poor sleep quality (β = -.18, p = .002). Depressive symptoms were positively associated with poor sleep quality (β = .34, p <.001).

**Figure 2:**
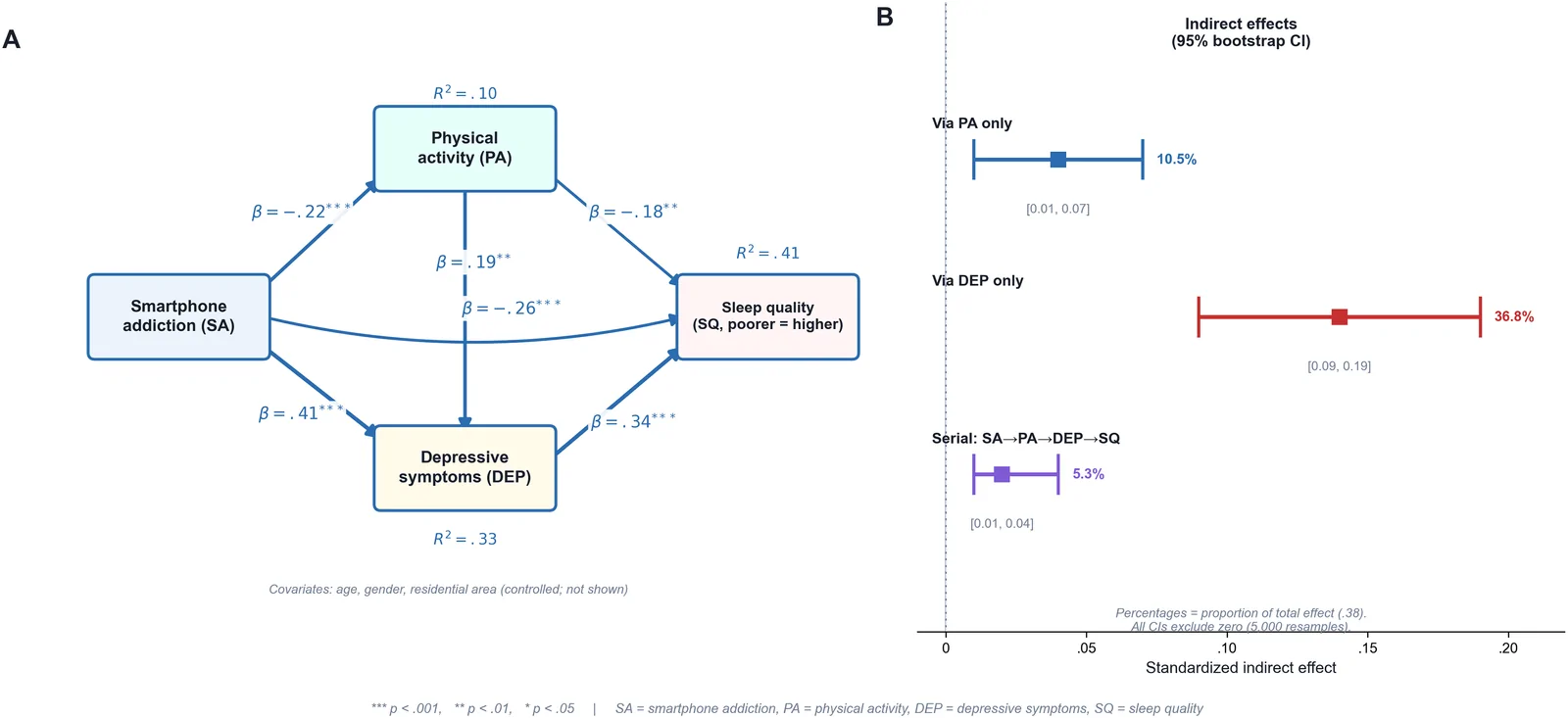
Standardized path coefficients of the final path model [Panel **(A)**] and standardized indirect effects with 95% bootstrap confidence intervals [Panel **(B)**]. SA, smartphone addiction; PA, physical activity; DEP, depressive symptoms; SQ, sleep quality. Solid arrows represent significant paths. Percentages above the bars indicate each indirect effect as a proportion of the total effect. *p <.05, **p <.01, ***p <.001.

The model explained 10.3% of the variance in physical activity, 33.4% of the variance in depressive symptoms, and 41.2% of the variance in sleep quality. The total effect of smartphone addiction on poor sleep quality was significant (β = .38, p <.001). After including the mediators, the direct effect remained significant (β = .19, p = .003), indicating partial mediation.

### 3.3 Mediation effects

Bias-corrected bootstrapping with 5,000 resamples was used to test the significance of the indirect effects. The results are summarized in **Table 2**. Three significant indirect pathways were identified. First, the indirect effect through physical activity alone was significant (point estimate = .04, SE = .02, 95% CI [.01,.07]), representing 10.5% of the total effect and 20.0% of the total indirect effect. Second, the indirect effect through depressive symptoms alone was significant (point estimate = .14, SE = .03, 95% CI [.09,.19]), representing 36.8% of the total effect and 70.0% of the total indirect effect. Third, the serial mediation pathway (smartphone addiction → low physical activity → depressive symptoms → poor sleep quality) was significant (point estimate = .02, SE = .01, 95% CI [.01,.04]), representing 5.3% of the total effect and 10.0% of the total indirect effect. Together, the mediators accounted for 52.6% of the total effect; all percentages reported above are expressed relative to the total effect unless otherwise indicated.

**Table 2:** Direct and indirect effects of smartphone addiction on sleep quality.

Pathway	Point estimate	SE	95% CI (LL)	95% CI (UL)
Total effect	.38	.04	.30	.46
Direct effect	.19	.06	.07	.31
Total indirect effect	.20	.04	.13	.28
Via physical activity only	.04	.02	.01	.07
Via depressive symptoms only	.14	.03	.09	.19
Serial (PA → DEP)	.02	.01	.01	.04

N = 612. CI, confidence interval; PA, physical activity; DEP, depressive symptoms. All estimates are standardized. 5,000 bootstrap resamples. Significant indirect effects have CIs that do not include zero. As proportions of the total effect (.38), the indirect effects via PA, via DEP, and the serial pathway represent 10.5%, 36.8%, and 5.3%, respectively; as proportions of the total indirect effect (.20), they represent 20.0%, 70.0%, and 10.0%, respectively.

## 4 Discussion

The present study tested a parallel and serial mediation model examining whether physical activity and depressive symptoms mediate the association between smartphone addiction and sleep quality in Chinese adolescents. The results supported all three hypothesized pathways, contributing to a more comprehensive understanding of the mechanisms through which excessive smartphone use may impact adolescent sleep.

First, we found that smartphone addiction was associated with reduced physical activity engagement, which in turn was associated with poorer sleep quality. This finding is consistent with the displacement hypothesis (5), which posits that time spent on smartphones may displace time available for physical activity. However, displacement is only one possible account of this association: the direction could also be reversed, such that adolescents who are less physically active turn to smartphones to fill leisure time, and third variables such as academic pressure or limited access to sports facilities could contribute to both smartphone overuse and low activity. Longitudinal and experimental designs are needed to adjudicate among these explanations. Reduced physical activity may impair sleep through multiple mechanisms, including reduced sleep drive, altered circadian rhythms, and decreased body temperature regulation (9, 10). The WHO (27) recommends that adolescents engage in at least 60 minutes of moderate-to-vigorous physical activity daily; however, the mean PAQ-A score in our sample (2.7 on a 5-point scale) suggests that many participants fell short of this guideline.

Second, smartphone addiction was associated with elevated depressive symptoms, which in turn were associated with poorer sleep quality. This finding aligns with growing evidence linking excessive screen time to increased depressive symptoms in adolescents (4, 13). Several mechanisms have been proposed, including reduced face-to-face social interaction, upward social comparison, cyberbullying, and disrupted sleep–wake cycles (3, 28). Depressive symptoms are well established as a strong predictor of sleep disturbances, as depression can alter sleep architecture, increase physiological arousal, and amplify cognitive rumination at bedtime (14).

Third, and most novel, the serial mediation pathway (smartphone addiction → reduced physical activity → elevated depressive symptoms → poor sleep quality) was significant. This pattern is consistent with a sequence of associations in which smartphone addiction is linked to lower physical activity, which in turn is associated with elevated depressive symptoms and, ultimately, poorer sleep quality. This finding extends the stress buffering hypothesis (16) to the digital context: when physical activity—the coping resource—is lower alongside smartphone overuse, the protective buffer against depressive symptoms may be weakened, and the association with sleep disturbance may be strengthened. From a self-determination theory perspective (17, 18), reduced physical activity may fail to satisfy basic psychological needs for autonomy, competence, and relatedness, thereby increasing vulnerability to depressive symptoms. At the same time, the magnitude of this serial indirect effect was small (point estimate = .02, representing 5.3% of the total effect and 10.0% of the total indirect effect). Although statistically reliable, its practical significance is modest, and it should not be over-interpreted as evidence of a dominant causal cascade; rather, it indicates that the physical activity–depression chain is one of several pathways linking smartphone addiction to sleep, and its temporal ordering cannot be established in the present cross-sectional data.

The practical implications of these findings, while provisional given the cross-sectional design, are worth noting. First, interventions targeting smartphone addiction could be complemented by physical activity promotion. School-based programs that encourage physical activity could, in principle, serve a dual purpose: reducing time available for excessive smartphone use and providing a buffer against depressive symptoms and sleep disturbances. Second, sleep hygiene education for adolescents could address smartphone use patterns and mental health alongside sleep-specific recommendations. Third, the identification of a serial pathway raises the possibility that multi-component interventions (addressing smartphone use, physical activity, and depressive symptoms together) might offer additive benefits, although this conjecture requires testing in randomized controlled trials before firm recommendations can be made.

Several limitations should be acknowledged. First, the cross-sectional design precludes causal inference. Although the hypothesized directions are theoretically motivated, all variables were measured at a single time point, and reverse or reciprocal relationships (e.g., poor sleep exacerbating smartphone use) cannot be ruled out; longitudinal and experimental studies are needed to confirm the temporal ordering of the observed associations. Second, all measures were self-reported, which may introduce recall bias and social desirability effects; the measures also differed in their recall periods (the PAQ-A covers the past 7 days, the DASS-21 the previous week, the PSQI the past month, and the SAS-SV has no specific timeframe), which may introduce temporal misalignment across the modeled pathways. Future studies could incorporate objective measures of smartphone use (e.g., screen time tracking applications) and physical activity (e.g., accelerometers). Third, the sample was recruited by convenience sampling from four schools in one city in central China, potentially limiting generalizability, and participants were nested within schools; because school-level clustering was not modeled, standard errors may be slightly underestimated if intraclass correlations were non-trivial, and multilevel approaches should be considered in future work. Fourth, the sample spanned grades 7 through 12 (ages 12–18), a wide developmental window during which smartphone use patterns, physical activity, and sleep change considerably; although age was statistically controlled, developmental heterogeneity within the sample may have masked age-specific pathways. Fifth, the Harman single-factor test used to assess common method bias is known to be insensitive; future studies should employ multiple data sources or procedural remedies. Sixth, only the depression subscale of the DASS-21 was administered, so the findings cannot speak to anxiety or stress, and other potential mediators such as anxiety, loneliness, or fear of missing out were not measured. Seventh, covariate selection was limited to age, gender, and residential area; residual confounding by unmeasured variables (e.g., socioeconomic status, academic pressure, chronotype) remains possible. Eighth, the SAS-SV cutoff values used to estimate the prevalence of smartphone addiction were developed in Korean samples, and their applicability to Chinese adolescents has not been firmly established; prevalence estimates should therefore be interpreted with caution. Ninth, the *a priori* power analysis relied on a regression-based approximation rather than a simulation-based power analysis for the full mediation model, so the study may have been underpowered to detect small indirect effects. Finally, the serial mediation effect was small (5.3% of the total effect), suggesting that other pathways not captured in the current model also contribute to the association between smartphone addiction and sleep quality.

The present findings should be interpreted in the context of the broader literature on digital stress and adolescent health. The concept of digital stress encompasses not only the quantity of screen time but also the psychological demands of constant connectivity, social comparison, and information overload (3). From this perspective, smartphone addiction may be both a correlate and a consequence of digital stress, creating a self-perpetuating cycle that is associated with poorer physical activity engagement, mental health, and sleep. Because only the depression subscale of the DASS-21 was administered, the present findings speak to depressive symptoms specifically and cannot be generalized to anxiety or stress, which are also integral components of digital stress. Future research should examine whether interventions targeting digital stress management (e.g., digital detox programs, mindfulness-based approaches) can break this cycle by reducing smartphone dependence and promoting healthier behavioral patterns. Additionally, the role of social support, which the stress buffering hypothesis identifies as another key coping resource (16), warrants investigation as a potential moderator of the observed pathways.

From a practical standpoint, the results suggest that school administrators and health educators could adopt an integrated approach to addressing adolescent sleep health. Rather than focusing solely on reducing screen time, interventions could incorporate structured physical activity programs that serve multiple functions: providing an alternative to smartphone use, promoting physical and mental well-being, and improving sleep. The finding that the pathway through physical activity represented 10.5% of the total effect, while that through depressive symptoms represented 36.8%, suggests that targeting mental health may have the largest association with sleep improvement, but combining mental health support with physical activity promotion may yield additive benefits. Furthermore, parents could be informed about the interrelated associations among smartphone addiction, physical activity, mental health, and sleep, and encouraged to establish household rules that promote balanced technology use and regular physical activity.

## 5 Conclusion

The present study demonstrates that smartphone addiction is associated with poor sleep quality among Chinese adolescents through both parallel and serial pathways involving physical activity and depressive symptoms. The serial mediation finding is consistent with the possibility that smartphone addiction is linked to poorer sleep in part through its association with reduced physical activity and elevated depressive symptoms, although the cross-sectional design precludes causal conclusions. These results highlight the potential value of promoting physical activity as a protective strategy in the digital age. Future longitudinal and intervention studies are needed to test the hypothesized temporal ordering and to evaluate the efficacy of multi-component interventions targeting smartphone use, physical activity, and mental health simultaneously.

## Data Availability

The raw data supporting the conclusions of this article will be made available by the authors, without undue reservation.
